# The Characteristics and Locking Process of Nonlinear MEMS Gyroscopes

**DOI:** 10.3390/mi11020233

**Published:** 2020-02-24

**Authors:** Yan Su, Pengfei Xu, Guowei Han, Chaowei Si, Jin Ning, Fuhua Yang

**Affiliations:** 1 Engineering Research Center for Semiconductor Integrated Technology, Institute of Semiconductors, Chinese Academy of Sciences, Beijing 100083, China; suyan16@semi.ac.cn (Y.S.); xupengfei@semi.ac.cn (P.X.); ningjin@semi.ac.cn (J.N.); 2 College of Materials Science and Opto-Electronic Technology, University of Chinese Academy of Sciences, Beijing 100049, China; 3 School of Electronic, Electrical and Communication Engineering, University of Chinese Academy of Sciences, Beijing 100049, China; 4 State Key Laboratory of Transducer Technology, Chinese Academy of Sciences, Beijing 100083, China; 5 State Key Laboratory of Superlattices and Microstructures, Institute of Semiconductors, Chinese Academy of Sciences, Beijing 100083, China

**Keywords:** micro-electro-mechanical system (MEMS), gyroscopes, nonlinearity, phase-locked loop (PLL), nonlinear PLL (NPLL)

## Abstract

With the miniaturization of micro-electro-mechanical system (MEMS) gyroscopes, it is necessary to study their nonlinearity. The phase-frequency characteristics, which affect the start-up time, are crucial for guaranteeing the gyroscopes’ applicability. Nevertheless, although the amplitude-frequency (A-f) effect, one of the most obvious problems in nonlinearity, has been well studied, the phase response of nonlinear gyroscopes is rarely mentioned. In this work, an elaborate study on the characteristics and locking process of nonlinear MEMS gyroscopes is reported. We solved the dynamic equation using the harmonic balance method and simulated the phase-locked loop (PLL) actuation process with an iterative calculation method. It was shown that there existed an apparent overhanging and multi-valued phenomenon in both the amplitude–frequency and phase–frequency curves of nonlinear gyroscopes. Meanwhile, it was ascertained by our simulations that the locking time of PLL was retarded by the nonlinearity under certain conditions. Moreover, experiments demonstrating the effect of nonlinearity were aggravated by the high quality factor of the drive mode due to the instability of the vibration amplitude. A nonlinear PLL (NPLL) containing an integrator was designed to accelerate the locking process. The results show that the start-up time was reduced by an order of magnitude when the appropriate integral coefficient was used.

## 1. Introduction

In the past decade, micro-electro-mechanical system (MEMS) gyroscopes, which are used for detecting the angular velocity of objects in inertial systems, have occupied a large market share in navigation control and consumer electronics due to their small size, low power consumption, and low cost [1,2,3]. The gyroscope consists of two orthogonal resonators, the drive mode, and the sense mode. The stability of the drive mode is a prerequisite for the normal operation of gyroscopes. Many performance metrics, including the scale factor, linearity, and bias instability, are affected by the stability of the drive mode. Meanwhile, the time for missile missions is usually below 180 seconds, while the flight time in tank applications is only 10 seconds [4,5]. Therefore, it is necessary to drive gyroscopes quickly and steadily to the operation state.

Until now, there are two primary actuation methods for MEMS gyroscopes: phase-locked loop (PLL) and self-oscillation [6,7,8]. The self-oscillation method is simple, but the absolute accuracy and stability of phase-shifting are critical to the high-precision frequency tracking. The PLL is an automatic control system that synchronizes the input and output phases. With a high loop gain, high-precision frequency tracking can be achieved. A traditional PLL is composed of a phase detector (PD), a loop filter (LF), and a voltage-controlled oscillator (VCO). Researchers usually give a block diagram of the PLL, but rarely mention the specific locking process. Therefore, it is necessary to find a method to accurately analyze the locking process when a gyroscope is actuated using a PLL. 

Meanwhile, the study of nonlinear gyroscopes has been significant in the last two decades due to the continuous size reduction [9,10]. The nonlinearity in MEMS gyroscopes has a large variety of sources. It mainly stems from intrinsic material effects, geometric nonlinearity, and electro-mechanical nonlinearity [11,12,13]. The harmfulness of nonlinearity in MEMS gyroscopes has been discussed in detail [13], including the phase noise, frequency stability, and hysteresis of the sweep frequency. Among the nonlinear features of gyroscopes, the most obvious one is the amplitude–frequency (A-f) effect, which means that the resonant frequency is related to the amplitude of the driving force. Because the gyroscope is an electro-mechanical device, the effect of nonlinearity on gyroscopes is ultimately reflected in the vibration characteristics. It is noteworthy that the quality factor of the drive mode (*Q_x_*) will affect the vibration response’s stabilization process [14]; therefore, its influence on nonlinearity will be discussed in this paper.

A cubic term of vibration displacement is introduced into the dynamic equation when the gyroscope displays nonlinear characteristics, in which case the drive mode of nonlinear gyroscopes can be modeled as a Duffing oscillator [15]. This so-called Duffing equation is solved via the harmonic balance method in this paper, and the corresponding amplitude response, as well as the phase response, are obtained. The stability of the closed-loop drive system in the presence of nonlinearity is demonstrated under certain characteristics [16]. For nonlinear gyroscopes, self-oscillation is not suitable due to the existence of the A-f effect. Therefore, it is necessary to study the PLL method to drive the nonlinear gyroscope quickly and stably. The drive mode of gyroscopes and the PLL constitute a closed-loop system, and the final operation point is the intersection of their phase–frequency curves. The length of the locking process is the duration of frequency oscillation from the initial frequency of PLL to the final operation frequency. Simulations in this paper show that the nonlinearity of a gyroscope will elongate the locking process under certain conditions. A large amount of work has been published to reduce the nonlinearity in MEMS gyroscopes [17,18]. Tatar et al. [18] experimentally indicated that a shaped finger design can cancel the softening nonlinearity by introducing a DC-voltage-controlled cubic hardening. In contrast to the structural improvement, another method is to optimize the PLL circuit itself. In this study, the phase–frequency curve of the PLL was changed by designing an integrator before the VCO to accelerate the locking process of nonlinear gyroscopes. The relevant simulations and experiments were performed to validate our design. There are three aspects to the contribution of the present work:

(1) The dynamic equation of a nonlinear gyroscope was solved using the harmonic balance method, and the corresponding amplitude–frequency, as well as phase–frequency curves, were mapped. Meanwhile, the effect of a high quality factor on linearity is also explained. 

(2) The iterative calculation was used to show the locking process when gyroscopes were actuated using a PLL. The corresponding simulations indicated that the nonlinearity would delay the gyroscope’s locking process under certain conditions. 

(3) A nonlinear PLL (NPLL) containing an integrator was designed. The simulation and experimental results showed that the improved locking process of nonlinear gyroscopes was accelerated, and the drive mode vibrated at the resonant frequency with maximum energy utilization.

## 2. Analysis and Methods 

### 2.1. The Working Principle and Characteristics of Gyroscopes

**Figure 1:**
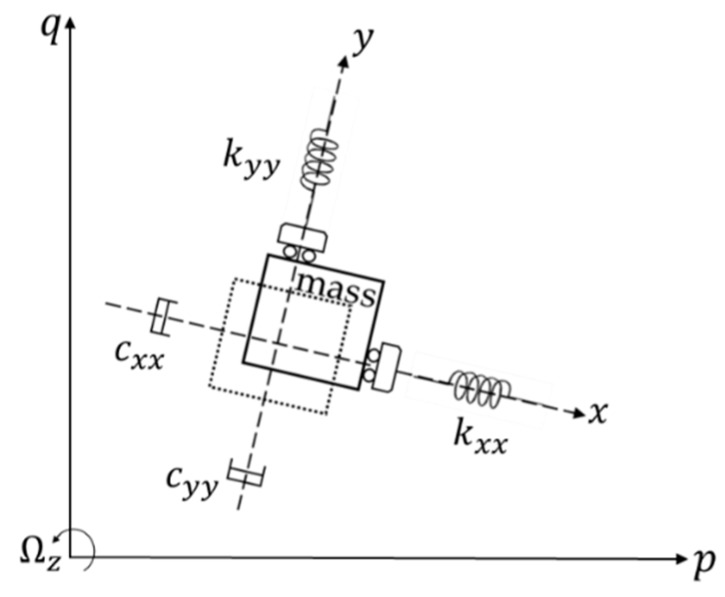
Gyroscope principle.

A typical MEMS gyroscope usually consists of mass blocks, anchor points, support beams, actuation combs, detection combs, feedback combs, and stiffness correction combs. It is an angular rate sensor based on the Coriolis effect in classical Newtonian mechanics, whose principle is that a moving object tends to continue vibrating in the same plane even if its support rotates. The Coriolis effect causes the object to exert a force perpendicular to the direction of motion on its support. Figure 1 shows the working principle.

In Figure 1, the dotted box and the solid box represent the initial position and new position of the mass block, respectively. The mass is forced to undergo a sinusoidal vibration along the *x*-axis. The stiffnesses of the folded support beams are disparate in different directions, the result of which means that the movements of the drive and sense frame do not affect each other. When the device experiences a rotation, a Coriolis displacement will be generated in the orthogonal direction (*y*-axis) of the original velocity. Therefore, the vibration of the gyroscope consists of a drive mode and a sense mode, in which the drive mode is along the *x*-axis and is used to generate a stable vibration, including frequency tracking and amplitude stabilization. The sense mode is along the *y*-axis and is used to extract the input angular rate by detecting the Coriolis displacement. Both the drive mode and sense mode can be regarded as a “spring-mass-damping” second-order vibration system. 

Ignoring the coupling between the two modes, the Coriolis force on the *x*-axis caused by input angular velocity, and the nonlinearity of drive mode, the dynamic equation is:

(1) $$m_{x}\ddot{x}+c_{xx}\dot{x}+k_{xx}=F_{x},$$

where *c_xx_*, *k_xx_*, *m_x_*, and *F_x_* are the damping coefficient, stiffness coefficient, effective mass, and driving force of the drive mode, respectively. *x*, $\dot{x}$ and $\ddot{x}$ are the displacement, velocity, and acceleration of the drive mode, respectively. *c_xx_* and *k_xx_* can be represented by other parameters and Equation (1) can be rewritten as:

(2) $$\omega_{x}={\left(\frac{k_{xx}}{m_{x}}\right)}^{\frac{1}{2}},$$

(3) $$Q_{x}=\frac{m_{x}\omega_{x}}{c_{xx}},$$

(4) $$\ddot{x}+\frac{\omega_{x}}{Q_{x}}\dot{x}+\omega_{x}{}^{2}x=F_{x}/m_{x},$$

where $\omega_{x}$ and $Q_{x}$ are the resonant angular frequency and the quality factor of the drive mode, respectively. $F_{x}$ changes as a sinusoidal function and can be expressed as $A_{f}\cos\omega_{d}t$. $A_{f}$ and $\omega_{d}$ are the amplitude and angular frequency of the driving force, respectively. The vibration displacement of the drive mode is calculated in Cao et al. [14] and is given as follows:

(5) $$\begin{array}{l} x=\frac{A_{f}/m_{x}}{\sqrt{{\left(\omega_{x}{}^{2}-\omega_{d}{}^{2}\right)}^{2}+\omega_{x}{}^{2}\omega_{d}{}^{2}/Q_{x}{}^{2}}}\cos(\omega_{d}t+\phi_{d})\\ +\frac{A_{f}\omega_{d}\omega_{x}/\left(m_{x}Q_{x}\right)}{{\left(\omega_{x}{}^{2}-\omega_{d}{}^{2}\right)}^{2}+\omega_{x}{}^{2}\omega_{d}{}^{2}/Q_{x}{}^{2}}e^{\frac{-\omega_{x}}{2Q_{x}}t}\cos(\sqrt{1-1/\left(4Q_{x}{}^{2}\right)}\omega_{x}t)\\ +\frac{A_{f}\omega_{d}(\omega_{x}{}^{2}/Q_{x}{}^{2}+\omega_{d}{}^{2}-\omega_{x}{}^{2})/m_{x}}{\omega_{x}\sqrt{1-1/\left(4Q_{x}{}^{2}\right)}\left[{\left(\omega_{x}{}^{2}-\omega_{d}{}^{2}\right)}^{2}+\omega_{x}{}^{2}\omega_{d}{}^{2}/Q_{x}{}^{2}\right]}e^{\frac{-\omega_{x}}{2Q_{x}}t}\sin\left(\sqrt{1-1/\left(4Q_{x}{}^{2}\right)}\omega_{x}t\right), \end{array}$$

where $\phi_{d}$ is the phase shift of the vibration displacement relative to the driving force. The analytical solution shows that the vibration of the drive mode consists of two transient components and a steady-state component. The amplitude of both transient components decays exponentially with time. Therefore, the amplitude of a high-$Q_{x}$-factor gyroscope is unstable during the starting oscillation process, and the oscillation time has a positive correlation with the $Q_{x}$ factor. An excitation experiment using a fixed-frequency signal was performed to measure the start-up process after power on. The experimental results are shown in Section 3.1. 

### 2.2. The Characteristics and PLL’s Locking Process of Nonlinear Gyroscopes and the Related Improvement

#### 2.2.1. The Frequency Characteristics of Nonlinear Gyroscopes

The dynamic equation of a MEMS gyroscope’s drive mode is:

(6) $$\ddot{x}+\delta \dot{x}+kx=A\cos\omega_{d}t,$$

where the parameters δ, k, and *A* control the amount of damping, the stiffness, and the amplitude of the periodic driving force, respectively. For gyroscopes, nonlinearity mainly comes from mechanical nonlinearity and electro-mechanical nonlinearity [13]. Mechanical nonlinearity affects the stiffness coefficient k [13]:

(7) $$k=k_{0}\left(1\;+\;k_{1}x\;+\;k_{2}x^{2}\;+\;\ldots \right).$$

$k_{1}$ is usually zero due to the symmetry of the structures. Therefore, the dynamic equation of the drive mode will induce a $k_{0}k_{2}x^{3}$ term, which is written as “$\beta x^{3}$.” In this case, the nonlinearity is called hardening (β > 0) and the amplitude–frequency curve bends to the right. 

Another common source of nonlinearity is the electro-mechanical nonlinearity [13,19]. The electrostatic force $f_{e}$ between two parallel plates can be expanded using the Taylor series about a system’s zero deflection point in the case of a push–pull architecture [19]:

(8) $$f_{e}=\overset{2n+1}{\underset{j=1}{\sum }}k_{j}x^{j}V_{dc}{}^{2},$$

where $V_{dc}$ is the biasing voltage. The even-order terms are canceled out due to symmetry of the exerted forces. We consider nonlinearities up to the third order; therefore, there is a term “$k_{3}x^{3}V_{dc}{}^{2}$” on the right side of the dynamic equation of drive mode. In this case, the nonlinearity is called softening (β< 0) and the amplitude curve bends to the left. Therefore, both mechanical nonlinearity and electro-mechanical will introduce a “$\beta x^{3}$” term. Therefore, the complete dynamic equation of a nonlinear gyroscope is:

(9) $$\ddot{x}+\delta \dot{x}+\alpha x+\beta x^{3}=A\cos\omega_{d}t,$$

where the parameters α and β control the amount of linear stiffness and nonlinearity, respectively. Equation (9) is a nonlinear, second-order differential equation, which is usually called the Duffing equation [18]. It is impossible to calculate the exact analytical solution for this equation. However, using the method of harmonic balance, we can derive two corresponding frequency response equations, which are approximate solutions to the Duffing equation. First, an approximate solution to the Duffing equation is sought of the form:

(10) $$x=a\cos(\omega_{d}t)+b\sin(\omega_{d}t)=Z\cos\left(\omega_{d}t+\phi_{d}\right),$$

(11) $$Z^{2}=a^{2}+b^{2},$$

(12) $$\tan\phi_{d}=-\frac{b}{a},$$

where $a\cos(\omega_{d}t)$ and $b\sin(\omega_{d}t)$ are two orthogonal components of the vibration displacement. Z is the amplitude of the vibration displacement. Applying these formulas to the Duffing equation while neglecting the superharmonics at 3$\omega_{d}$, the frequency response equations can be obtained. The specific mathematical calculations are shown in Appendix A.

(13) $$[{\left(\omega_{d}{}^{2}-\alpha -\frac{3}{4}\beta Z^{2}\right)}^{2}+{\left(\delta \omega_{d}\right)}^{2}]Z^{2}=A^{2}$$

(14) $$Z\omega_{d}\delta =-A\sin\phi_{d}$$

Equation (13) is an implicit equation, whose variables are A and *Z*. Equation (14) is an implicit equation of three variables, and the extra one is *ϕ_d_*. We selected the same parameters from Sohanian-Haghighi and Davaie-Markazi [20] (δ=0.1, α=1) for the calculation and simulation. The numerical solutions of these implicit equations under specific conditions are mapped in Section 3.2.1. In fact, due to the presence of nonlinearity, the amplitude–frequency and phase–frequency curves of a nonlinear gyroscope are no longer monotonic. Many papers only discuss the amplitude–frequency characteristics at different driving forces, which is called the “A-f” effect [13,18]. However, the influence of nonlinearity on the phase characteristics is rarely discussed. This paper explains the effect of the phase–frequency characteristics of a nonlinear gyroscope on the locking process. 

#### 2.2.2. The Effect of Nonlinearity on PLL’s Locking Process

In this paper, the phase characteristics of the drive mode are significant because it and the phase characteristics of PLL determine the locking process of a nonlinear gyroscope. Intuitively, the final working frequency of the drive mode is the frequency of the intersection of the gyroscope’s phase–frequency and PLL’s phase–frequency curves. Mathematically it is the solution of the following two equations:

(15) $$Z\omega_{d}\delta =-A\sin\phi_{d},$$

(16) $$\omega_{d}=\omega_{0}+k_{d}\cos\phi_{d},$$

where $\omega_{0}$ and $k_{d}$ are the initial angular frequency and linear gain factor of the VCO, respectively. Equation (16) is the phase–frequency relationship of a traditional PLL. This PLL consists of a multiplier, a low-pass filter (LPF), and a VCO. The output of an LPF was assumed to be $\cos\phi_{d}$ with a unit gain for the sake of discussion. A swept frequency excitation experiment was conducted to demonstrate the nonlinearity of the gyroscopes developed by our group. The figure below shows the experimental result of the gyroscope developed by our group.

**Figure 2:**
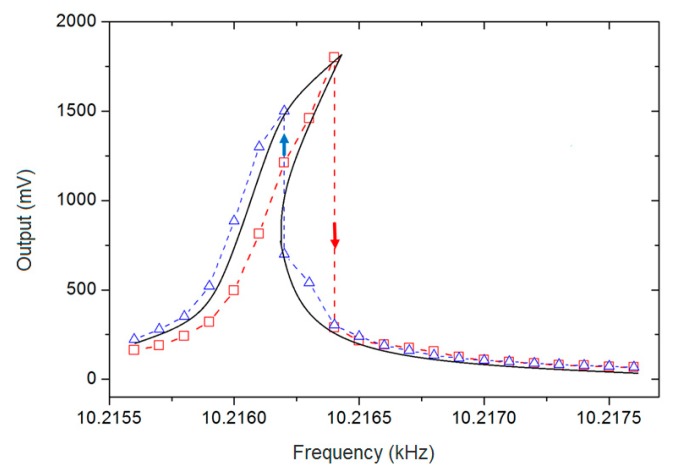
Output of drive mode in sweeping frequency excitation.

The output in Figure 2 represents the output voltage of the drive mode. Notice that the resonant frequencies measured by sweeping the frequency up (red dots and dash line) and sweeping the frequency down (blue dots and dash line) were different. The black line is the synthesized amplitude–frequency curve (bending to the right) according to the experimental results and it demonstrates the positive polarity of β. 

Section 2 in Tiwari and Candler [13] analyzes that the experimental measurement of Duffing nonlinearity has an obvious phenomenon, which is where the branch is unstable and approaching it from one stable branch causes the resonator to jump to the other stable branch. Notice that the output changes of our gyroscope have this jump phenomenon in both the red and blue lines, which further verifies the nonlinearity.

To verify the effect of nonlinearity on the PLL’s locking process, two contrastive simulations with different *β* were performed based on the iterative method. The PLL used in the simulations was the same. Specifically, the PLL exported a signal with an initial frequency to drive the gyroscopes to generate the vibration displacement, which had a phase shift relative to the excitation signal. The PD discriminated the phase shift and generated an error signal, which impelled the VCO to generate a new excitation signal with a changed frequency. This process was iterated to the final operation state or divergent state, in which the gyroscope’s vibration was unstable. The detailed simulations are shown in Section 3.2.2.

#### 2.2.3. The Design of a Nonlinear PLL

**Figure 3:**
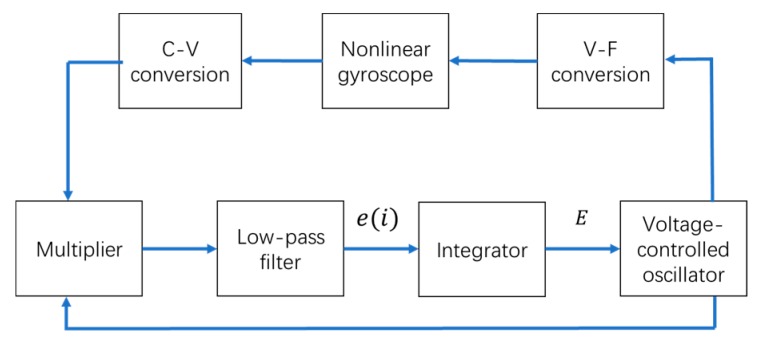
The system composed of a nonlinear gyroscope and a nonlinear phase-locked loop (NPLL). C-V: capacitance-voltage, V-F: voltage-force.

The above analysis indicates the traditional PLL is deficient for a nonlinear gyroscope. One improvement is to shorten the locking process, and the other is to ensure the gyroscope vibrates at the resonant frequency, in which case, the gyroscope has the maximum amplitude and energy utilization and a −90° phase shift between the driving force and the vibration displacement. In this study, an integrator was designed into the traditional PLL to achieve the improvements mentioned above. The novel PLL is called a nonlinear PLL (NPLL). The closed-loop system was composed of an NPLL and a nonlinear gyroscope is shown in Figure 3. 

When the closed-loop system is stable, it means the frequency of the VCO’s output signal is constant or perturbed within a small range. In this case, the output of the LPF is zero or has perturbations around zero; otherwise, the frequency of the VCO’s output signal is changed because of the existence of an integrator. Therefore, the phase shift between the driving force and the vibration displacement is −90° such that the drive mode vibrates at the resonant frequency. The phase shift of the capacitance-voltage (C-V) conversion is ignored here. The mathematical form of the integrator is:

(17) $$E=K_{c}\sum e{\left(i\right)}^{n},$$

where E and $K_{c}$ are the output of the integrator and the integration coefficient, respectively. e(i) is the output of the LPF. n is the power of the integral term. By adding an integrator, the phase–frequency curve of the PLL is changed to shorten the locking process of a nonlinear gyroscope, and this is implemented via the frequency of the VCO’s output signal being quickly iterated to the resonant frequency. The corresponding simulations and experiments were performed to verify the validity of this integrator design.

## 3. Results and Discussion

### 3.1. The Start-Up Oscillation Process of MEMS Gyroscopes

**Figure 4:**
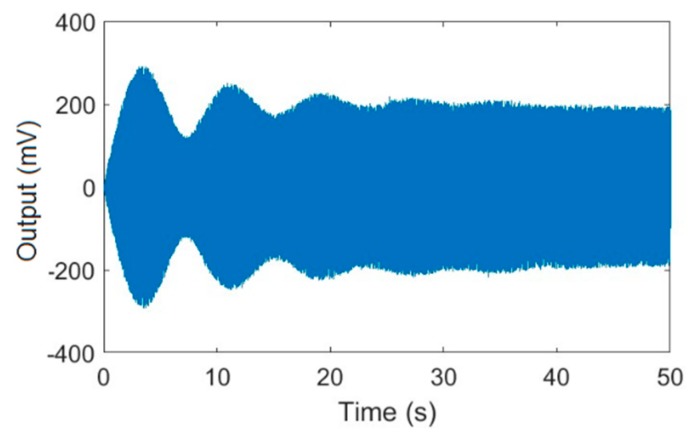
The output of drive mode during the start-up oscillation process.

For this section, the same experiments were conducted for a gyroscope with a higher $Q_{x}$ factor and a gyroscope with a lower $Q_{x}$ factor, both of which were developed by our group and have nonlinear characteristics. Here we list the experimental data of the gyroscope with a higher $Q_{x}$ factor. An excitation experiment with a fixed-frequency signal was performed to measure the start-up oscillation process of the gyroscope. Figure 4 shows the output of the drive mode over time. 

The result shows that the vibration amplitude oscillated during the start-up process, which was due to the continuous attenuation of the transient response. It took about 40 seconds to stabilize the vibration amplitude, which demonstrated that the $Q_{x}$ factor was very high. 

**Figure 5:**
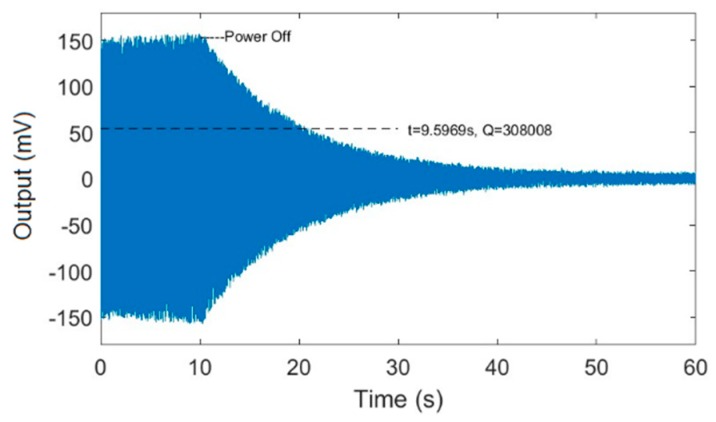
The output of the drive mode over time after power-off.

A power-off experiment was performed to calculate the exact $Q_{x}$. The specific experimental method was described in References [21,22]. Figure 5 shows the output of the drive mode over time after power-off. 

The result shows that the vibration amplitude of the drive mode decayed with time after power-off. The $Q_{x}$ factor is the embodiment of the system energy loss; therefore, the $Q_{x}$ factor can be calculated using the attenuation of the vibration amplitude. The formula for calculating the $Q_{x}$ factor value given in Zhang et al. [21] is:

(18) $$Q_{x}=\pi f_{x}\frac{1}{\lambda },$$

where $f_{x}$ is the resonant frequency of the drive mode. $\frac{1}{\lambda }$ is the relaxation response time after power-off, and the specific value was about 9.6 s. The calculated value of $Q_{x}$ factor was about 300,000. We also repeatedly measured the lower-$Q_{x}$-factor gyroscope, whose start-up stabilization process and power-off decay process was rapid, and the value of the $Q_{x}$ factor was about 50,000. The two gyroscopes were used to design comparative experiments that explained the effect of the quality factor on the locking process of gyroscopes with nonlinearity.

### 3.2. The Simulation and Experimental Results of Nonlinear Gyroscopes

#### 3.2.1. The Frequency Characteristics of Nonlinear Gyroscopes

**Figure 6:**
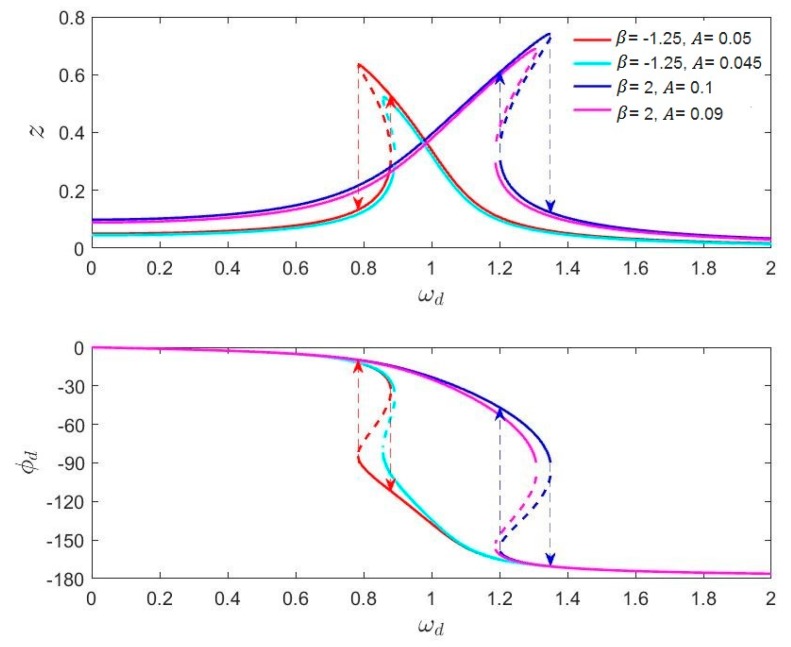
The amplitude and phase response of nonlinear gyroscopes.

The amplitude and phase response of nonlinear MEMS gyroscopes under different driving forces were mapped by solving Equations (13) and (14) and are shown in Figure 6.

The results show that the nonlinearity of MEMS gyroscopes caused an overhanging and a multi-valued phenomenon in the amplitude–frequency and phase–frequency curves. Meanwhile, the magnitude of the driving force caused these curves to change. Figure 6 clearly shows the “A-f” effect of nonlinear gyroscopes, which has been mentioned in many papers. The characteristic of the phase–frequency curve changing with the driving force is significant, especially for the locking process of a high-$Q_{x}$ nonlinear gyroscope.

#### 3.2.2. The PLL’s Locking Process of Nonlinear Gyroscopes

**Figure 7:**
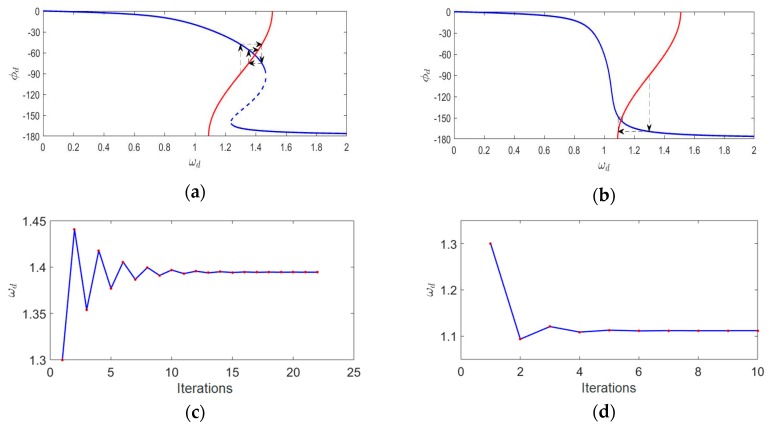
(**a**) The locking process diagram of a nonlinear gyroscope (*β* = 3.3), (**b**) the locking process diagram of a gyroscope with poor nonlinearity (*β =* 0.1), (**c**) the $\omega_{d}$ with iterations for a nonlinear gyroscope (*β =* 3.3), and (**d**) the $\omega_{d}$ with iterations for a gyroscope with poor nonlinearity (*β =* 0.1)

An identical PLL was used to drive a nonlinear gyroscope and a gyroscope with poor nonlinearity in turn. According to the iterative method mentioned above, the process of each iteration was recorded in detail until the gyroscopes were finally stabilized at the operation point. We assumed that the system reached a steady state when the angular frequency difference before and after an iteration was less than 0.00005. The phase–frequency relationship of the PLL was $\omega_{d}=1.3+0.21\cos\phi_{d}$. Figure 7 shows the detailed simulation results.

**Figure 8:**
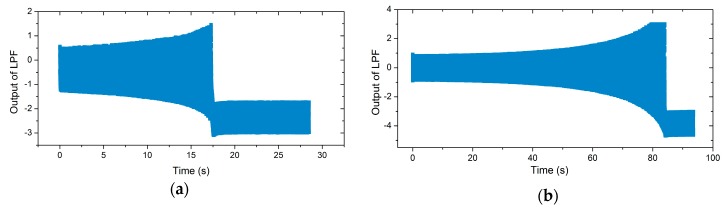
(**a**) The output of the low-pass filter (LPF) in the phase-locked loop (PLL) for the lower-$Q_{x}$-factor gyroscope over time, and (**b**) a contrastive experiment for the higher-$Q_{x}$ -factor gyroscope

Figure 7a,b shows the illustration of the iterative method in the locking process of the gyroscopes. The blue line is the phase–frequency curve of the gyroscopes, while the red line is the phase–frequency curve of the PLL. The intersection of these two curves is the final operation point. The results show that the region of frequency variation during the locking process was different in the presence of distinct nonlinearity. Figure 7c,d shows the $\omega_{d}$ with iterations in both cases. Compared with the gyroscope with poor nonlinearity, the iterative number of the nonlinear gyroscope’s locking process was greater, which meant a longer locking time. Therefore, the locking time of the PLL was retarded by the nonlinearity under certain conditions. An experiment was conducted to show the actual locking process of a nonlinear gyroscope, which was the lower $Q_{x}$ factor one mentioned in Section 3.1. The experimental result is shown in Figure 8a. For a high-$Q_{x}$ nonlinear gyroscope, the unstable vibration amplitude due to the transient response during the start-up process could be simplified to the varying steady-response amplitude generated by an unstable excitation signal while ignoring the transient response. Therefore, according to the effect of the magnitude of the driving force on the phase characteristics shown in Figure 6, the phase–frequency curve of a high-$Q_{x}$-factor gyroscope with nonlinearity during the start-up process was unstable. This unsteadiness affected the nonlinear gyroscope’s locking process. A contrastive experiment was conducted using the higher-$Q_{x}$-factor gyroscope mentioned in Section 3.1. Figure 8b shows the experimental results.

In actual tests, when the output of the LPF oscillated within a fixed range (the rectangular section to the right of the timeline in Figure 8a,b), the drive mode generated an observable sinusoidal waveform. This means the operation frequency of the PLL’s output signal was slightly perturbed in practice when the drive mode was stable. The locking process refers to the system from power on to this slight disturbance state. The experimental results show that the frequency locking process of the nonlinear gyroscope was >10 s in duration, and a higher $Q_{x}$ factor made the locking time longer, which is consistent with the previous analysis. 

#### 3.2.3. The Simulation and Experimental Results of the NPLLs

**Figure 9:**
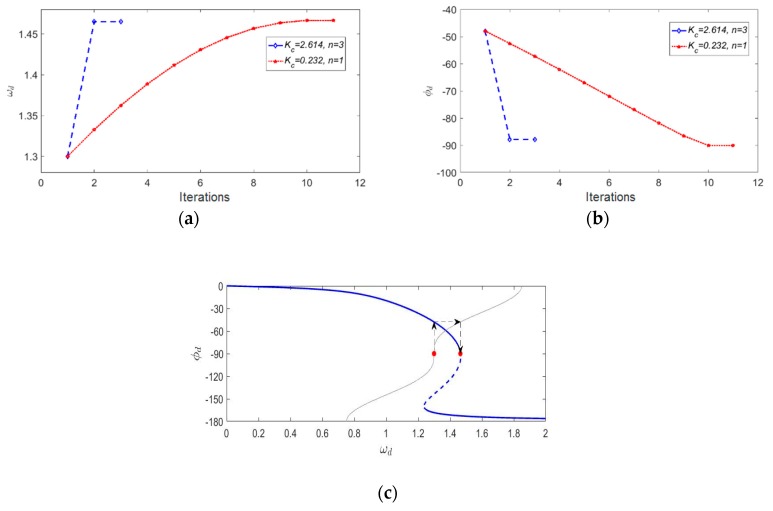
(**a**) The $\omega_{d}$ with iterations using NPLLs with different parameters, (**b**) the $\phi_{d}$ with iterations using NPLLs with different parameters, and (**c**) the locking process diagram of a nonlinear gyroscope using an NPLL (*β =* 3.3,$\;K_{c}$ = 2.614, n = 3).

Equation (17) shows that both the integration coefficient and the power of the integral term affect the design of an NPLL. When n is an even number, the output of the integrator is positive, which means the frequency of VCO’s output signal is always greater than the initial frequency, which means the NPLL cannot complete the locking process when the resonant frequency of drive mode is less than the initial frequency. Therefore, we chose the value of n to be 1 and 3 to simulate the locking process of an NPLL. When n was different, in order to realize the fast iteration of the frequency of the VCO’s output signal to the resonant frequency, the calculated $K_{c}$ was also different. Therefore, simulations of different parameters were implemented. Here, a nonlinear gyroscope (*β =* 3.3) was used in the simulation. Figure 9 shows the simulation results in detail.

**Figure 10:**
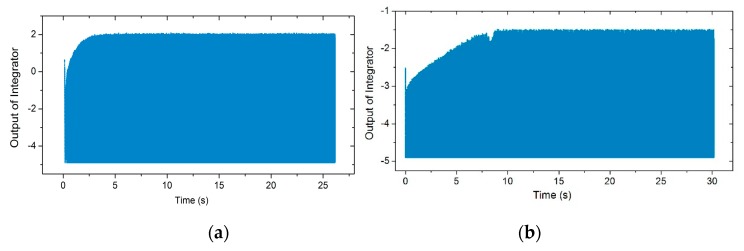
(**a**) The integrator’s output for the lower-*Q_x_*-factor gyroscope over time using an NPLL, and (**b**) the integrator’s output for the higher-*Q_x_*-factor gyroscope over time using an NPLL.

Figure 9a shows that for NPLLs with different parameters, the numbers of iterations was reduced compared to Figure 7c. Therefore, the frequency locking process of nonlinear gyroscopes could be accelerated by adding an integrator into a traditional PLL. Figure 9b shows that the phase shift between the driving force and the vibration displacement was −90° under different parameters, which means that the gyroscope vibrated at the resonant frequency. Figure 9c shows the locking process under specific parameters, in which the thin gray line is the phase–frequency curve of the NPLL during the first iteration. Note that the intersection of the gray line and the blue line is not the final operation point because the gray line changes with time because of the integrator. In fact, the red dots are the operation points in the iterative process. By designing an appropriate coefficient $K_{c}$, the operation frequency converged to the vicinity of the resonant frequency after one iteration, and the locking process was completed in this case. Meanwhile, two experiments were performed to validate the integrator design using the lower-$Q_{x}$- and the high-$Q_{x}$-factor gyroscopes mentioned in Section 3.1. The corresponding experimental result is shown in Figure 10. 

It can be seen that the frequency locking time of the lower-$Q_{x}$ and the higher-$Q_{x}$ nonlinear gyroscopes using the NPLL was about 3 s and 9 s, respectively. For the PLL and NPLL, the output of the LPF and the output of the integrator were used to control the VCO, respectively. Therefore, these two different parameters were used to compare the locking time of the working frequency. Compared with the traditional PLL excitation method, the frequency locking time using the NPLL method was reduced by about an order of magnitude, which verified the effectiveness of the NPLL design. 

**Figure 11:**
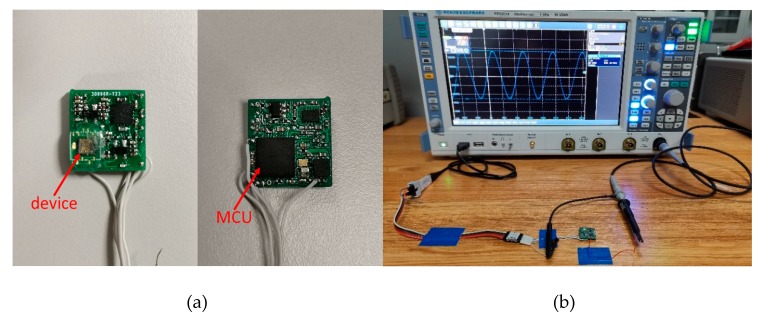
(**a**) The gyroscope system and (**b**) the complete test system.

A mixed analog/digital circuit was designed to complete the overall test. A printed circuit board (PCB) was fabricated to mount with the test circuit, and the gyroscope was fixed after being packaged in a wafer level. The PCB was divided into the obverse and the reverse. Besides the interface circuit of gyroscopes and power supply, the whole signal processing was based on a digital algorithm in a microcontroller unit (MCU). The test system and gyroscope system are shown in Figure 11. 

The test system was composed of the nonlinear gyroscope device, peripheral circuit, oscilloscope, and power supply. The whole gyroscope system was powered by a USB interface. The wave in the oscilloscope was a pure sine wave, which indicates that the gyroscope with nonlinearity was locked at the working frequency successfully. 

## 4. Conclusions

This study investigated the characteristics and driving process of nonlinear gyroscopes. First, the amplitude and phase response of nonlinear gyroscopes were mapped by solving the dynamic equation using the harmonic balance method. Then, the locking process of gyroscopes was analyzed, and an iterative calculation method was proposed to obtain precise changes in frequency and phase during the locking process. The simulation results showed that the region of frequency variation was different and the locking process was lengthened in the presence of a distinct nonlinearity. Corresponding experiments were conducted to show the actual locking process of a nonlinear gyroscope and verify that the high $Q_{x}$ factor had a negative effect on the locking time. Finally, an integrator was added to the traditional PLL to artificially change its phase–frequency curve. Simulation results indicated that the locking process of a nonlinear gyroscope could be completed in the initial iterations by designing an appropriate integration coefficient. The experimental results show that the locking time was shortened by an order of magnitude using the NPLL. Simultaneously, the gyroscope vibrated at its resonant frequency with maximum energy utilization.

## Appendix A

The dynamic equation of a nonlinear gyroscope is $\ddot{x}+\delta \dot{x}+\alpha x+\beta x^{3}=A\cos\omega_{d}t,$. Using the method of harmonic balance, an approximate solution to the Duffing equation is sought of the form:

$$x=a\cos(\omega_{d}t)+b\sin(\omega_{d}t)=Z\cos\left(\omega_{d}t-\phi \right),\mathrm{with}Z^{2}=a^{2}+b^{2}\mathrm{and}\;\tan\phi =\frac{b}{a}.$$

Application in the Duffing equation leads to:

$$\begin{array}{l} \left(-\omega_{d}{}^{2}a+\omega_{d}\delta b+\alpha a+\frac{3}{4}\beta a^{3}+\frac{3}{4}\beta ab^{2}-A\right)\cos\left(\omega_{d}t\right)+\left(-\omega_{d}{}^{2}b-\omega_{d}\delta a+\alpha b+\frac{3}{4}\beta b^{3}+\frac{3}{4}\beta ba^{2}\right)\\ \sin(\omega_{d}t)+(\frac{1}{4}\;\beta a^{3}-\frac{3}{4}\beta ab^{2})\cos(3\omega_{d}t)+(-\frac{1}{4}\beta b^{3}+\frac{3}{4}\;\beta ba^{2})\sin(3\omega_{d}t)=0. \end{array}$$

Neglecting the superharmonics at $3\omega_{d}$, the two terms $\cos\left(\omega_{d}t\right)$ and $\sin\left(\omega_{d}t\right)$ must be zero. As a result:

$$-\omega_{d}{}^{2}a+\omega_{d}\delta b+\alpha a+\frac{3}{4}\beta a^{3}+\frac{3}{4}\beta ab^{2}-A=0\;(A1),\;-\omega_{d}{}^{2}b+\omega_{d}\delta a+\alpha b+\frac{3}{4}\beta b^{3}+\frac{3}{4}\beta ba^{2}=0\;(A2).$$

Squaring both equations and adding leads to the amplitude-frequency response:

$$[{\left(\omega_{d}{}^{2}-\alpha -\frac{3}{4}\beta Z^{2}\right)}^{2}+{\left(\delta \omega_{d}\right)}^{2}]Z^{2}=A^{2}.$$

By using Equation (A1) divided by Zcosϕ and Equation (A2) divided by Zsinϕ, and subtracting both new equations leads to the phase response:

$$Z\omega_{d}\delta =A\sin\phi .$$
